# Transition to Psychosis in Individuals at Clinical High Risk: Meta-analysis

**DOI:** 10.1192/j.eurpsy.2023.797

**Published:** 2023-07-19

**Authors:** G. Salazar De Pablo, J. Radua, I. Bonoldi, V. Arienty, F. Besana, A. Cabras, A. Catalan, P. Fusar-Poli

**Affiliations:** 1 Child and Adolescent Psychiatry, Institute of Psychiatry, Psychology & Neuroscience, Department of Psychosis Studies, King’s College London, London, United Kingdom; 2 Hospital Clinic Barcelona, Barcelona, Spain; 3 Department of Psychosis, King’s College London, London, United Kingdom; 4 University of Pavia, Pavia; 5 University of Rome, Rome, Italy; 6 Department of Psychosis Studies, IoPPN, King’s College London; 7 Early Psychosis: Interventions and Clinical-detection (EPIC) Lab, Institute of Psychiatry, Psychology & Neuroscience, Department of Psychosis Studies, King’s College London, London, United Kingdom

## Abstract

**Introduction:**

Estimating the current likelihood of transitioning from a clinical high risk for psychosis (CHR-P) to psychosis holds paramount importance for preventive care and applied research.

**Objectives:**

Our aim was to quantitatively examine the consistency and magnitude of transition risk to psychosis in individuals at CHR-P.

**Methods:**

This meta-analysis is compliant with Preferred Reporting Items for Systematic Reviews and Meta-analyses (PRISMA) and Meta-analysis of Observational Studies in Epidemiology (MOOSE) reporting guidelines. PubMed and Web of Science databases were searched for longitudinal studies reporting transition risks in individuals at CHR-P.

Primary effect size was cumulative risk of transition to psychosis at 0.5, 1, 1.5, 2, 2.5, 3, 4, and more than 4 years’ follow-up, estimated using the numbers of individuals at CHR-P transitioning to psychosis at each time point. Random-effects meta-analysis were conducted.

**Results:**

A total of 130 studies and 9222 individuals at CHR-P were included. The mean (SD) age was 20.3 (4.4) years, and 5100 individuals (55.3%) were male.

The cumulative transition risk was 9% (95% CI = 7-10% k = 37; n = 6485) at 0.5 years, 15% (95% CI = 13-16%; k = 53; n = 7907) at 1 year, 20% (95% CI = 17%-22%; k = 30; n = 5488) at 1.5 years, 19% (95% CI = 17-22%; k = 44; n = 7351) at 2 years, 25% (95% CI, 21-29%) at 2.5 years, 25% (95% CI = 22-29%; k = 29; n = 4029) at 3 years, 27% (95% CI = 23-30%; k = 16; n = 2926) at 4 years, and 28% (95% CI = 20-37%; k = 14; n = 2301) at more than 4 years.

Meta-regressions showed that a lower proportion of female individuals (β = -0.02; 95% CI, -0.04 to -0.01) and a higher proportion of brief limited intermittent psychotic symptoms (β = 0.02; 95% CI, 0.01-0.03) were associated with an increase in transition risk. Other predictors were not statistically significant (p **>** 0.05).

Heterogeneity across the studies was high (I2 range, 77.91% to 95.73%).

**Conclusions:**

In this meta-analysis, 25% of individuals at CHR-P developed psychosis within 3 years. Transition risk continued increasing in the long term. Extended clinical monitoring and preventive care may be beneficial in this patient population.

**Disclosure of Interest:**

None Declared

